# The Effect of Primer Choice and Short Read Sequences on the Outcome of 16S rRNA Gene Based Diversity Studies

**DOI:** 10.1371/journal.pone.0071360

**Published:** 2013-08-19

**Authors:** Jonas Ghyselinck, Stefan Pfeiffer, Kim Heylen, Angela Sessitsch, Paul De Vos

**Affiliations:** 1 Laboratory of Microbiology, Department of Biochemistry and Microbiology, Ghent University, Ghent, Belgium; 2 Department of Health and Environment, Bioresources Unit, AIT Austrian Institute of Technology GmbH, Tulln, Austria; Institute for Genome Sciences, University of Maryland School of Medicine, United States of America

## Abstract

Different regions of the bacterial 16S rRNA gene evolve at different evolutionary rates. The scientific outcome of short read sequencing studies therefore alters with the gene region sequenced. We wanted to gain insight in the impact of primer choice on the outcome of short read sequencing efforts. All the unknowns associated with sequencing data, i.e. primer coverage rate, phylogeny, OTU-richness and taxonomic assignment, were therefore implemented in one study for ten well established universal primers (338f/r, 518f/r, 799f/r, 926f/r and 1062f/r) targeting dispersed regions of the bacterial 16S rRNA gene. All analyses were performed on nearly full length and *in silico* generated short read sequence libraries containing 1175 sequences that were carefully chosen as to present a representative substitute of the SILVA SSU database. The 518f and 799r primers, targeting the V4 region of the 16S rRNA gene, were found to be particularly suited for short read sequencing studies, while the primer 1062r, targeting V6, seemed to be least reliable. Our results will assist scientists in considering whether the best option for their study is to select the most informative primer, or the primer that excludes interferences by host-organelle DNA. The methodology followed can be extrapolated to other primers, allowing their evaluation prior to the experiment.

## Introduction

Next generation sequencing (NGS) platforms have allowed microbiologists to gain new insights in microbial ecology [1]. Through high-throughput amplicon sequencing of specific target genes such as the 16S rRNA gene, researchers have been enabled to get a glimpse of microbial communities in environments of interest [2]. However, a number of steps, which include sampling, DNA extraction and PCR, may hamper the objective of obtaining results truly representing the environment studied [3]. One essential aspect demanding careful consideration is primer choice. Particular genes, such as the 16S rRNA gene in bacteria, contain regions that have evolved at different evolutionary rates, and as such the scientific outcome may vary with the gene region sequenced [4], [5], [6], [7]. The 16S rRNA gene consists of fast evolving, structural parts that are defined as variable regions V1-V9, and that allow the identification of bacteria. The term ‘hypervariable region’ was designated to those regions of the 16S rRNA gene of which the evolutionary rate exceeds the mean evolutionary rate of all nucleotides in the molecule [8]. However, there are clear differences in base heterogeneity and phylogenetic discriminatory power between the different regions [9], [10]. The important issue of primer universality has been discussed previously [11], [12], [13]. The 16S rRNA gene contains several conserved stretches that are shared amongst almost all known bacteria [13], [14], and that are used to develop universal primers. However, the coverage rates of such primers differ with the location of their target in the 16S rRNA gene. Online matching tools such as SILVA Test Probe [12] and RDP probe match [15] have been specifically developed to address this problem. Furthermore, Berry et al. [16] have reported biases introduced with barcode-tagging of primers that translate into less reproducible data sets, while Wu and colleagues [17] extensively mentioned the problems of preferential amplification.

The analysis of bacterial communities associated with hosts, such as plants and weeds, may be hampered by the interference of host organelles. In order to efficiently extract the bacterial DNA pool from a host matrix, bacteria ought to be released from the host matrix prior to, or during DNA extraction. This often requires a vigorous DNA extraction, which will also release organelle DNA. As a consequence, microbial community studies that are based on high-throughput amplicon sequencing of the 16S rRNA gene may experience problems due to the undesired co-amplification of mitochondrial 18S and chloroplast 16S rRNA. As plant organelles sometimes outnumber bacterial cells, it is desirable to specifically amplify prokaryotic genes. The 799 primer [18] could be of special interest for studying microbial communities obtained from host matrices. The 799 primer is known to allow the exclusion of host derived chloroplast sequences by targeting the bacterial 16S rRNA gene, while failing to target the gene in chloroplasts [18]. Moreover, if used in the forward direction, and in combination with a well-chosen reverse primer, a mitochondrial amplicon will be generated that is larger than the corresponding bacterial amplicon [18], which allows their separation by gel electrophoresis.

Several studies have focused on coverage rates of primers targeting different regions of the bacterial 16S rRNA gene [12], [13], while others have analyzed the phylogenetic information that is contained within short reads [10]. Schloss et al. [4] analyzed the effects of different data processing approaches on alpha- and beta-diversity for different regions of the bacterial 16S rRNA gene, while others studied the results of taxonomic assignments with reads generated from different 16S rRNA gene targeting primers [5], [6], [19], [20]. However, uniformity between each of these studies, which provided very useful insights into the advantages and limitations of the short read sequencing approach, is lacking. Therefore, it can be difficult to e.g. be aware of the phylogenetic information that is contained within reads that were generated from a primer with a well documented coverage rate, and what the effect of its use will be on OTU richness and taxonomic assignment. To account for this shortcoming, we implemented the unknowns that are associated with primer choice, i.e. primer coverage rate, OTU-richness, taxonomic assignment, and phylogeny, in one study for ten different primers, including the 799 primer, targeting dispersed regions of the bacterial 16S rRNA gene. Our motivation was to get a clear picture of the intrinsic information loss that is associated with sequencing of short reads compared to their parent nearly full length (NFL) sequences covering the V1-V9 variable regions. The results of this study will allow researchers to select primers based on the objectives of their research, and will assist them with the interpretation of their results. Moreover, the approach followed will allow scientists to evaluate new primers before using them in short read sequencing based experiments.

## Materials and Methods

### Primer selection and coverage rate

For this study, we chose well established universal 16S rRNA gene primers (Table 1), each of which target conserved stretches between the hypervariable regions V1–V9 of the 16S rRNA gene that were described by Van de Peer et al [8]. Primer coverage rates were calculated both at the domain and phylum level by using the tool “SILVA Test Probe” [12]. SILVA [21] provides chimera checked, aligned sequences which form todaýs standard SSU rRNA database. The primers and their reverse complements were matched against the non redundant (NR) SILVA SSU Ref dataset 113 [22], allowing no mismatches.

**Table 1:** Primer sequences and their domain specific coverage rates.

Primer^a^	Sequence (5′–>3′)	*E. coli* Position	Coverage (%)^b^				Reference
			Eukarya	Bacteria	Archaea	Total	
338r	GCTGCCTCCCGTAGGAGT	355–338	-	88,4	-	75,6	Suzuki (1996) [44]
							
518r	ATTACCGCGGCTGCTGG	542–518	88,3	85,1	0,4	82,2	Muyzer (1993) [45]
							
799f	AACMGGATTAGATACCCKG	781–799	-	78,5	71,7	69,4	Chelius & Triplett (2001) [18]
							
926f	AACTCAAAGGAATTGACGG	908–926	-	77,4	-	65,7	Lane (1991) [46]
							
1062r	CTCACRRCACGAGCTGAC	1081–1064	-	89,5	2,4	77,1	Allen (2005) [47]

a , Primer names according to first description; primer names indicate both position and direction.

b , According to SILVA SSU Ref 113 NR database.

### Selection of sequences and generation of the nearly full length library

To obtain a practicable but representative subset of the complete SILVA SSU reference dataset, NFL sequences were selected from the NR SILVA SSU reference database 102 [21]. The database in question contains ∼262 000 sequences that were chimera and quality checked, and redundancy filtered with the UCLUST tool [23]. In the frame of ‘The All Species Living Tree Project’ (LTP) [24], [25], a Maximum-Likelihood (ML) tree was constructed with RaxML [26] containing all UCLUST quality checked sequences. This allowed the display of the whole database in a tree format in the ARB software package [27]. We used this tree as a baseline for sequence selection, and thus for the construction of the practicable but representative sequence subset. In ARB, all eukaryotic and archaeal entries were removed, and the remaining bacterial tree was screened for phylogenetically distinct bacterial clades. Within each clade all except the entry containing the longest sequence were removed. Ideally, clades would contain members of the same genus, so that representatives per clade would represent the group of type strains within that clade. However, reality is different, as a number of genera are very closely related based on their 16S rRNA gene sequences. As a result, intrageneric phylogenetic distances within some genera sometimes exceed intergeneric distances between closely related genera (many genera of the *Enterobacteriaceae* for instance). In such cases, one sequence was selected per clade. Similarly, a number of bacterial genera such as for instance *Bacillus* and *Pseudomonas*, are known to harbour a high intrageneric diversity, containing distinct phylogenetic lineages. For such genera, sequences of several members of one genus were selected. For clades that only contained sequences from uncharacterized cultivation-independent sequence data, one full length, high quality 16S rRNA sequence entry was kept. The resulting tree contained 1186 16S rRNA gene sequences instead of the initial 262 000, while the original SSU Ref 102 LTP tree's branching pattern and phylogenetic distances were conserved. All 1186 sequences were exported in a fasta file. The end-points of all sequences were trimmed with the MEGA 5 software [28] as to obtain maximum overlap between the sequences. Subsequently, the library was analyzed in RAxML v7.3.2 to exclude identical sequences and gap-only characters in the alignment. As a consequence, the dataset was further reduced to 1175 sequences. All sequences of the NFL library contained the V1-V9 variable regions of the bacterial 16S rRNA gene.

### Generation of short read libraries

Ten short read (SR) libraries were constructed in MEGA 5 [28]; one library for each of the primers analyzed (Table 1). To do so, the NFL library was used as a seed by first locating the respective primers in the NFL library, and then trimming the sequences 280 bp upstream and downstream of the start of each primer (conform to unidirectional sequencing). The length of 280 bp for our SR libraries was based on suggestions made by Schloss and Quince. Although 454 amplicon sequencers generate reads with an average length of 400–700 bp, most quality checked sequences dońt exceed 280 bp due to quality assignments by leading packages Mothur [29] and QIIME [30]. Conversely, other NGS platforms, such as the Illumina sequencers, are now capable of generating longer reads. Therefore, the length of 280 bp, which was applied in this study, makes the results obtained applicable for a variety of NGS sequencers. After trimming primer sequences, libraries were ready for downstream analyses.

### Generation of short read and full length 16S rRNA gene trees

Each of the libraries was imported in RAxML v7.3.5 and a ML search was performed with the gamma parameter [31], in combination with rapid bootstrapping, which uses the CAT approximation [32]. The substitution model used was GTR. Bootstrapping was performed with 500 replicates. The command line used for the tree search was the following: raxmlHPC-PHTREADS-SSE3 –T <number of processors> -fa -m GTRGAMMA -N <replicates> -x <seed1> -p <seed2> -s <filename> -n <outputfile>. The best scoring ML tree was exported in newick format. Patristic distances, which are defined as the sum of the branch-lengths in the shortest path connecting a pair of taxa in a phylogenetic tree, were calculated for all pairs of taxa within the tree [10].

### Branch length based comparison of phylogenetic trees

#### The Pearson correlation between branch lengths of a pair of phylogenetic trees

In order to calculate the correlation between two ML trees, patristic distances between corresponding pairs of sequences in each of the trees were made into a tuple, which formed the coordinate of a point in a plot. This was performed for all pairs of sequences in each of the trees being compared. For each plot, the Pearson correlation was calculated and used as one measure to study the phylogenetic relation between two regions of the bacterial 16S rRNA gene. In order to present the data in a graph, branch-length distances were normalized to a maximum value of one and were ordered for the NFL tree. For each NFL distance interval of 0.01 we calculated the averages and standard deviations of corresponding patristic distances in the SR tree. Averaged NFL distances (over a 0.01 distance interval) and corresponding averaged SR distances were then plotted in a graph, and the standard deviations on the averaged SR distances were superimposed (as error bars) on the chart.

#### The degree of fit between a pair of phylogenetic trees using the vCEED approach

Patristic distance matrices were generated from the ML trees by using the PHYLOCOM software [33]. Distance matrices for each of the trees under comparison were used as inputs for the vCEED script that was written in Matlab by Choi and colleagues [34]. Using a distance matrix as an input, each sequence is mapped to a Euclidean space via metric multidimensional scaling (MDS). This produces a multidimensional plot in which each point represents one sequence (or taxon) within the phylogenetic tree (e.g. the NFL tree). The same procedure is then repeated for a second distance matrix, representing the phylogenetic tree we want to compare to the first one (e.g. the SR tree). Subsequently, one embedded point pattern is superimposed on the other and the degree of fit is calculated. The degree of fit is expressed by the weighted Root Mean Square Deviation (wRMSD). A decreasing wRMSD indicates an increasing degree of fit, and thus a higher similarity between trees. In addition, regions of high similarity as well as incongruent regions between the trees can be identified.

### Topology based comparison of phylogenetic trees

#### The Robinson Foulds distance between a pair of phylogenetic trees

The Robinson Foulds (RF) metric [35] was used to compare topologies of a pair of unrooted phylogenetic trees. It counts the number of bipartitions that occur in one tree but not in the other. The lower the RF value, the more similar both trees are with respect to tree topology. The weighted Robinson Foulds (WRF) metric, however, takes into account the bootstrap support values of the bipartitions instead of looking at their presence or absence only [36]. A bipartition with a bootstrap value of 0.6 counts 0.6 instead of 1, and as such the WRF metric penalizes less for lower supported bifurcations. Similarly, another metric was calculated that was derived from the WRF metric, and which we will refer to as WRF2. WRF2 not only includes the support value on each unique bipartition, but additionally includes the differing bootstrap support values of shared bipartitions. This provides additional information on the topological distance between a pair of trees. For this study, the RF and both WRF distances were calculated using RAxML v.7.4.2. Gui [26], [37].

#### Sliding window analysis on the nearly full length alignment

A sliding window analysis was performed with RAxML v7.3.5 to supplement the RF calculations between NFL and SR based trees. It tests for each sequence within the NFL library where the taxon would be placed in the best NFL tree, using only data contained within a sliding window of a given size. For our analysis, the size of the sliding window corresponded with the length of the short read sequences, i.e. 280 bp. After replacing the taxon in the tree based on the information contained within the sliding window, the software measures the distance in terms of nodes to the original placement. Hence, the sliding window analysis expresses the distance between the original NFL tree and the NFL tree that was modified according to the information that would be available if only short sequences were considered.

### The Pearson correlation between pairwise distances in a pair of sequence libraries and the effect on OTU richness

Pairwise distances were calculated between all pairs of sequences in each sequence library with RAxML v7.3.2. Pairwise distances between corresponding pairs of sequences in each of two libraries under comparison were made into a tuple, which then formed the coordinate of a point in a plot. For each plot, the Pearson Correlation was calculated. To present the data graphically, the same binning step was followed as for the branch-length distance correlation plots. To study the effect of pairwise distances altering with the region of the 16S rRNA gene sequenced on α-diversity, OTU richness was calculated for each SR library and for the NFL library. OTU richness was calculated using the Mothur v1.27.0 software [29] with the average neighbor clustering algorithm (i.e. UPGMA) and a hard cutoff [38]. Results obtained from the SR libraries were compared with results obtained from the NFL library by calculating the ratio of the number of OTUs obtained with each SR library to the number of OTUs obtained with the NFL library.

### Taxonomic assignment of sequences

*In silico* generated reads and the NFL sequences were assigned taxonomically using the Mothur v1.27.0 software, using the classify.seqs() tool. The RDP v9 training set [39] was used as a reference database. The bootstrap cutoff for assigning a sequence to a specific taxon was set at 80% based on suggestions made by Schloss.

## Results

### Primer Coverage Rate

With a total coverage rate of 82.2%, primer 518f/r showed the highest coverage amongst all primers investigated. The high value obtained was not only due to a high coverage within the domain Bacteria, but also due to a high coverage of eukaryotic 16S rRNA sequences (Table 1). This non-specificity, however, should be taken into consideration for bacterial community sequencing in many habitats, as it could cause contamination with eukaryotic 16S rRNA gene sequences. Primer 799f/r covered 78.5% of bacterial and 71.7% of archaeal sequences in the database. Primers 338f/r, 926f/r and 1062f/r showed almost no homology with sequences within the domains Eukarya and Archaea, which makes them almost exclusive for Bacteria.

**Figure 1:**
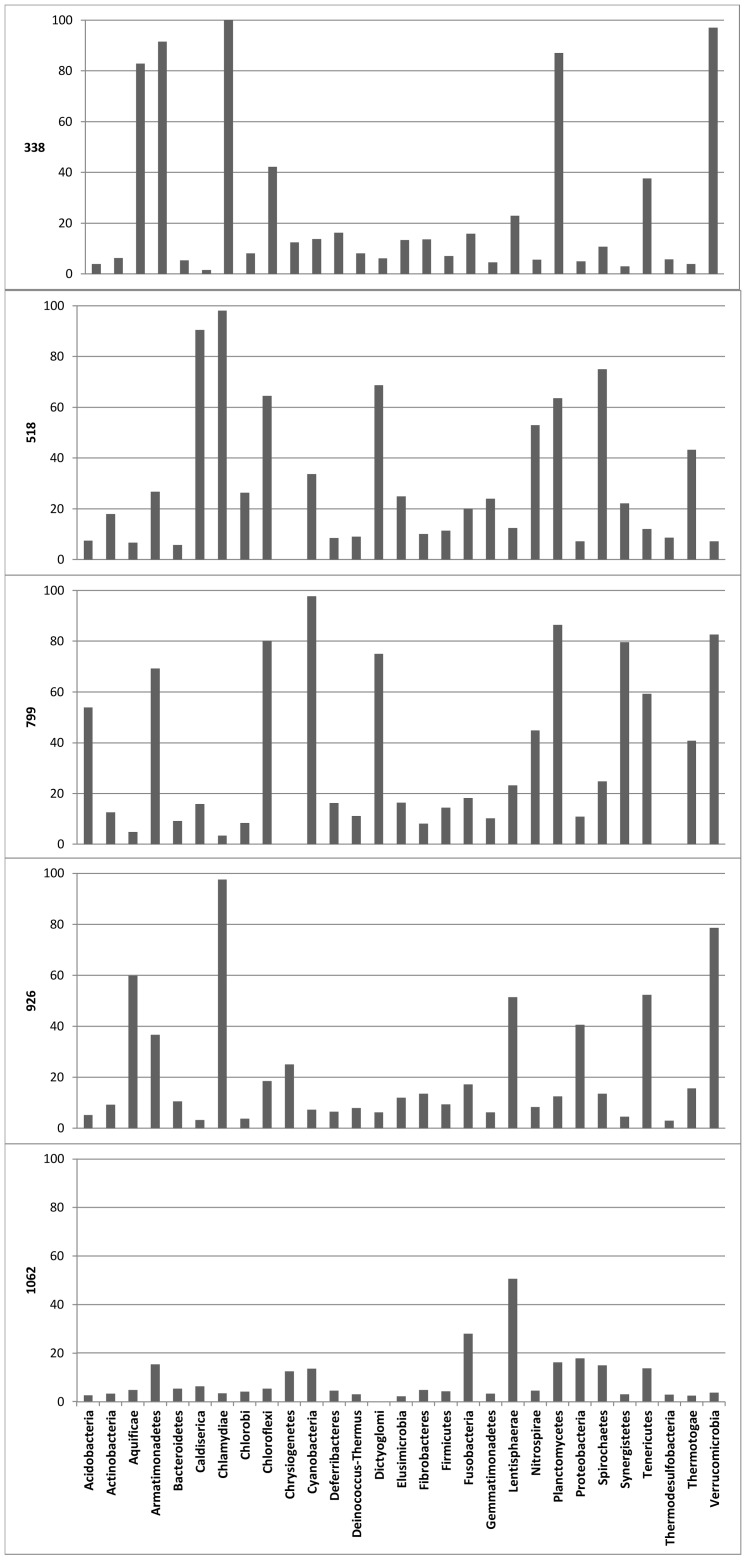
Percentage of non-coverage rates in 29 classified bacterial Phyla for the primers analyzed in this study. Non coverage rates were calculated based on the coverage values in the SILVA SSU Ref 113 NR database, using SILVA Test Probe with zero mismatches allowed.

Because total coverage rates bias towards large bacterial phyla such as the *Proteobacteria* and *Firmicutes*, non-coverage rates were calculated per phylum (Fig. 1). Non-coverage rates reflect the percentage of sequences that will not be covered by the primer investigated. Of the better represented phyla in the database, primer 799f/r was found to discriminate against almost all sequences of *Cyanobacteria*, against about 80% of *Planctomycete*s and *Verrucomicrobia* and against more than 50% of *Acidobacteria*. As chloroplasts are classified within the phylum *Cyanobacteria*, primer 799f/r can be considered to be of special interest for host-associated bacterial community studies. The lowest total coverage rate that was observed for the 926f/r primer (Table 1) seemed to be attributed to a low coverage of proteobacterial 16S rRNA gene sequences (Fig. 1). The highest total coverage rate in Bacteria was attributed to primer 1062f/r; its non-coverage rate did not exceed 40% in any of the phyla studied (Fig. 1). The non-coverage rates of primers 338f/r and 518f/r were generally low for the best represented phyla in the database. However, they were found to discriminate against specific taxonomic groups such as the *Verrucomicrobia* (Fig. 1).

### Phylogenetic content of short reads

Jeraldo et al. [10] reasoned that the branch length based correlation between trees generated from different tree searches on the same library can be used as a measure for the amount of phylogenetic information contained in a SR. A high Pearson Correlation will be obtained if sequences that are found to be closely together in the SR(1) tree are also found to be closely together in the SR(2) tree. Correlation values close to zero indicate the opposite, i.e. that sequences positioned closely together in the SR(1) tree are not necessarily found to be closely together in the SR(2) tree, meaning that the tree generated has high uncertainty with respect to branch lengths. Low correlations thus indicate that the information within the reads is too limited to calculate unequivocal branch lengths for a given sequence library, and as such is insufficient to solve the ML problem. To gain more insight in this matter, we calculated the correlation between two trees generated from the same library for the different libraries investigated. Since full length 16S rRNA gene sequences are the benchmark for constructing phylogenies [40], it was expected that the Pearson Correlation between different tree searches for NFL sequences would be the maximum correlation possible. However, the correlation between two tree searches from one NFL sequence library was 0.93 (Table 2) instead of the theoretically expected value of 1.00. This can be explained by the fact that ML trees are calculated using a heuristic method, and therefore there is no guarantee that the tree calculated best represents the sequence data, and thus is the best tree. Representation of sequence data in a phylogenetic tree which is based on heuristics is prone to uncertainties in tree structure, and therefore different tree searches for one and the same sequence library will unavoidably lead to differences in tree structure to some extent. Moreover, the random order in which sequences are added to a maximum parsimony starting tree in RAxML [41] is likely to generate several different starting trees for every new analysis that is started [42], again having implications for the “best tree”. Regardless, as the construction of ML trees from sequence data can only be as good as the phylogenetic information which it is generated from (i.e. the sequence data), we expect that the correlation between trees from different tree searches will be higher as more information is contained within the read. Surprisingly, a higher correlation was observed between two trees that were generated from the same 518f library (i.e. 0.97 (Table 2)). However, as explained above, ML is an approximation and there is no guarantee that the NFL tree calculated best represents the sequence data. As such, the possibility exists that the true NFL tree was ‘overlooked’. It is possible, although difficult to tell, that increasing the number of NFL starting trees during the ML calculation process would have resulted in higher correlations between trees obtained from different tree searches. The search for the best-known ML tree would in that case have started at different points in the vast search space and would have followed different trajectories, thus increasing chances of obtaining ML trees with higher likelihood values. Another possibility is that the initial sampling (two trees on the NFL alignment and two trees on the SR alignment) was too small, and that the higher correlation obtained for 518f reads happened by chance. This considered, we decided to generate five trees for all SR libraries investigated, and three for the NFL library. Table 2 shows a correlation of 0.98 between NFL(1) and NFL(3), which shows that our assumption was true and also confirms the upper-limit statement made earlier. Table 2 also shows that the high correlation values were maintained with a higher number of tree searches for the 518f library. Still, the upper-limit of 0.98 was not reached; correlation values ranged from 0.93 to 0.97 (coefficient of variation 0.015). This clearly shows that any tree constructed from the 518f reads is very robust with respect to patristic distances. Similarly, high correlations were obtained and maintained for different tree searches from 799r reads (coefficient of variation 0.019) (Table 2). These results indicate that any tree constructed from libraries targeting the V4 region of the bacterial 16S rRNA gene (i.e. 799r and 518f) is very stable with respect to branch-lengths. The V6-targeting 1062r read library on the other hand, showed the lowest correlation between trees generated from different tree searches, indicating its low reproducibility and thus phylogenetic content.

**Table 2:** Overview of research parameters that were used to measure the phylogenetic information contained within short read sequences and the OTU richness calculated from each library.

Libraries^a^	Variable region		PC patristic^b^		wRMSD^c^	RF^d^	WRF1^e^	WRF2^f^	RF-WRF1	RF-WRF2	OTU 0.01 cutoff^g^	OTU 0.02 cutoff^g^	OTU 0.03 cutoff^g^
NFL(1) vs NFL(2)	V1-V9		0.928		0.0098	585.3	121.07	155.98	462.93	428.02	-	-	-
NFL(1) vs NFL(3)			0.979		0.0041								
NFL(2) vs NFL(3)			0.943		0.0091								
338f(1) vs 338f(2)	V3		0.799		0.0135	1260.6	92.97	118.42	1167.63	1142.18	0.86	0.87	0.89
338f(1) vs 338f(3)			0.697		0.0182								
338f(1) vs 338f(4)			0.767		0.0156								
338f(1) vs 338f(5)			0.911		0.0098								
338f(2) vs 338f(3)			0.858		0.0143								
338f(2) vs 338f(4)			0.821		0.0137								
338f(2) vs 338f(5)			0.819		0.0139								
338f(3) vs 338f(4)			0.685		0.0178								
338f(3) vs 338f(5)			0.789		0.0157								
338f(4) vs 338f(5)			0.802		0.0154								
338r(1) vs 338r(2)	V2		0.846		0.0141	1359	85.17	110.33	1273.83	1248.67	0.82	0.84	0.84
338r(1) vs 338r(3)			0.851		0.014								
338r(1) vs 338r(4)			0.735		0.0191								
338r(1) vs 338r(5)			0.828		0.0138								
338r(2) vs 338r(3)			0.914		0.01								
338r(2) vs 338r(4)			0.642		0.0193								
338r(2) vs 338r(5)			0.828		0.0123								
338r(3) vs 338r(4)			0.699		0.0194								
338r(3) vs 338r(5)			0.826		0.0129								
338r(4) vs 338r(5)			0.729		0.0175								
518f(1) vs 518f(2)	V4		0.97		0.0062	1033.8	92.76	122.92	941.04	910.88	0.79	0.79	0.81
518f(1) vs 518f(3)			0.969		0.0059								
518f(1) vs 518f(4)			0.949		0.0077								
518f(1) vs 518f(5)			0.956		0.007								
518f(2) vs 518f(3)			0.952		0.0076								
518f(2) vs 518f(4)			0.931		0.0086								
518f(2) vs 518f(5)			0.934		0.0084								
518f(3) vs 518f(4)			0.937		0.0082								
518f(3) vs 518f(5)			0.942		0.0075								
518f(4) vs 518f(5)			0.956		0.0074								
518r(1) vs 518r(2)	V3		0.905		0.0112	1245.6	91.78	118.82	1153.82	1126.78	0.86	0.85	0.87
518r(1) vs 518r(3)			0.66		0.0206								
518r(1) vs 518r(4)			0.957		0.0069								
518r(1) vs 518r(5)			0.871		0.0117								
518r(2) vs 518r(3)			0.66		0.0201								
518r(2) vs 518r(4)			0.892		0.0115								
518r(2) vs 518r(5)			0.813		0.0151								
518r(3) vs 518r(4)			0.653		0.0211								
518r(3) vs 518r(5)			0.661		0.0213								
518r(4) vs 518r(5)			0.839		0.013								
799f(1) vs 799f(2)	V5		0.888		0.0106	1300.2	85.86	112.82	1214.34	1187.38	0.67	0.61	0.59
799f(1) vs 799f(3)			0.821		0.013								
799f(1) vs 799f(4)			0.941		0.0095								
799f(1) vs 799f(5)			0.941		0.0092								
799f(2) vs 799f(3)			0.914		0.0096								
799f(2) vs 799f(4)			0.822		0.0126								
799f(2) vs 799f(5)			0.817		0.013								
799f(3) vs 799f(4)			0.741		0.0155								
799f(3) vs 799f(5)			0.74		0.0159								
799f(4) vs 799f(5)			0.929		0.0084								
799r(1) vs 799r(2)	V4		0.92		0.0098	1143.6	99.29	128.54	1044.31	1014.06	0.81	0.77	0.79
799r(1) vs 799r(3)			0.91		0.0116								
799r(1) vs 799r(4)			0.89		0.0118								
799r(1) vs 799r(5)			0.95		0.0108								
799r(2) vs 799r(3)			0.95		0.0083								
799r(2) vs 799r(4)			0.93		0.0088								
799r(2) vs 799r(5)			0.92		0.0109								
799r(3) vs 799r(4)			0.93		0.0096								
799r(3) vs 799r(5)			0.92		0.0106								
799r(4) vs 799r(5)			0.91		0.0111								
926f(1) vs 926f(2)	V6		0.871		0.0129	1423	103.08	127.38	1319.92	1295.62	0.81	0.77	0.79
926f(1) vs 926f(3)			0.841		0.0145								
926f(1) vs 926f(4)			0.858		0.0161								
926f(1) vs 926f(5)			0.93		0.0118								
926f(2) vs 926f(3)			0.836		0.0132								
926f(2) vs 926f(4)			0.847		0.0157								
926f(2) vs 926f(5)			0.851		0.0132								
926f(3) vs 926f(4)			0.82		0.0173								
926f(3) vs 926f(5)			0.863		0.0136								
926f(4) vs 926f(5)			0.849		0.0165								
926r(1) vs 926r(2)	V5	0.857		0.0136		1228.4	86.39	113.04	1142.01	1115.36	0.73	0.69	0.7
926r(1) vs 926r(3)		0.87		0.016									
926r(1) vs 926r(4)		0.819		0.0143									
926r(1) vs 926r(5)		0.783		0.0151									
926r(2) vs 926r(3)		0.884		0.014									
926r(2) vs 926r(4)		0.924		0.0082									
926r(2) vs 926r(5)		0.812		0.0143									
926r(3) vs 926r(4)		0.769		0.0162									
926r(3) vs 926r(5)		0.844		0.0155									
926r(4) vs 926r(5)		0.729		0.015									
1062f(1) vs 1062f(2)	V7&8	0.95		0.0078		1212.6	75.51	102.48	1137.09	1110.12	0.68	0.64	0.6
1062f(1) vs 1062f(3)		0.88		0.0105									
1062f(1) vs 1062f(4)		0.9		0.0105									
1062f(1) vs 1062f(5)		0.93		0.0082									
1062f(2) vs 1062f(3)		0.87		0.0107									
1062f(2) vs 1062f(4)		0.91		0.0103									
1062f(2) vs 1062f(5)		0.9		0.0097									
1062f(3) vs 1062f(4)		0.78		0.0147									
1062f(3) vs 1062f(5)		0.89		0.0106									
1062f(4) vs 1062f(5)		0.84		0.0129									
1062r(1) vs 1062r(2)	V6	0.742		0.0164		1432.8	107.86	130.78	1324.94	1302.02	0.79	0.82	0.84
1062r(1) vs 1062r(3)		0.708		0.0179									
1062r(1) vs 1062r(4)		0.776		0.0152									
1062r(1) vs 1062r(5)		0.832		0.0163									
1062r(2) vs 1062r(3)		0.792		0.0155									
1062r(2) vs 1062r(4)		0.817		0.0145									
1062r(2) vs 1062r(5)		0.77		0.017									
1062r(3) vs 1062r(4)		0.698		0.0173									
1062r(3) vs 1062r(5)		0.83		0.0139									
1062r(4) vs 1062r(5)		0.722		0.0172									

a , NFL = Nearly Full Length.

b , PC =  Pearson Correlation.

c , wRMSD =  Weighted Root Mean Square Deviation.

d , RF =  averaged Robinson Foulds distances between five best ML trees.

e , WRF1 =  averaged Weighted Robinson Foulds distances between five best ML trees based on the sum of the supports of the unique bipartitions.

f , WRF2 =  averaged Weighted Robinson Foulds distance between five best ML trees based on the sum of the supports of the unique bipartitions plus the difference of support values amongst the shared bipartitions.

g , the ratio of the number of OTUs obtained with each short read library to the number of OTUs obtained with the nearly full length library.

Since comparing phylogenetic trees based on correlations between patristic distances is known to have its weaknesses [34], we strengthened our study by additionally applying the recently published vCEED approach [34]. A statistically significant negative correlation was found between results obtained with the vCEED approach (in terms of wRMSD), and those obtained with the Pearson Correlation method for comparisons of trees obtained from different tree searches on the same library (R = −0.93, p<0.0005). Similar to the Pearson Correlation approach, the highest degree of fit was found for NFL(1) vs NFL(3). Amongst the SR libraries, the highest degree of fit was observed for the 518f library, followed by the 799r and 1062f libraries. Supporting the observations obtained with the Pearson Correlation approach, the averaged wRMSD and the corresponding coefficients of variation were slightly lower for 799r reads than for 1062f reads (i.e. 0.0103 versus 0.0106, with coefficients of variation being 0.113 and 0.191 respectively) indicating its higher phylogenetic content. The V6 targeting 1062r read library again showed the largest variation among tree searches, which reflects its rather low phylogenetic content.

### Conservation of tree topology with different tree searches

To answer the question whether differences in branch length conservation amongst the different SR libraries investigated can be extrapolated to conservation of the tree's branching pattern, differences in topologies between trees generated from different tree searches on each SR library were calculated. Still, topological accuracy of a phylogenetic tree is not only a function of sequence length. The required sequence length to reach a given topological accuracy also depends on tree height, deviation from ultrametricity and the number of taxa included in the analysis [43]. Unweighted RF distance calculations showed that the 518f SR trees had the most consistent tree topology, followed by 799r and 1062f reads. Still, the RF distance was around two times higher than the RF distance between trees from different tree searches on the NFL library. The most variant tree topology was calculated for trees generated from the 1062r library, which confirmed the results obtained with patristic distances (Table 2).

The difference between RF and WRF values for a given tree comparison provides insight in the nature of differences in tree topology [10]. If the WRF value approximates the RF value, differences mainly occur in high-supported sub-trees, while a WRF value that is much lower than the corresponding RF value indicates that differences mainly occur on less supported bipartitions. Comparing tree topology conservation of the 518f and 799r tree sets with tree topology conservation of the 1062f tree set indicated that the topologies of the former were more conserved than topologies between trees generated from the 1062f library (Table 2). However, if penalized for the lower supported clades, trees generated from the 1062f library were more consistent with respect to tree topology conservation. Therefore, differences between the trees generated from different trees searches on the 518f and 799r SR libraries seem to occur on better supported branches than for trees that were generated from the 1062f SR library.

### Do SR reflect NFL phylogeny?

#### With respect to patristic distances

**Figure 2:**
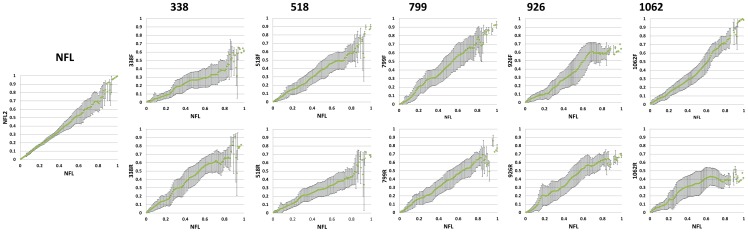
The Pearson Correlation between corresponding patristic distances in trees generated from nearly full length (x-axis) and short read libraries (y-axis) for the different primers investigated. Patristic distances were normalized to a maximum value of one prior to plotting.

The Pearson Correlation between corresponding patristic distances in trees generated from NFL and SR libraries was used to investigate if a read can be used to infer 16S rRNA gene based phylogeny. The correlation plots (Fig. 2) show that with the exception of the 1062r read library, there seemed to be no significant deviation from a straight line behavior, which is reflected by the correlation values given in Table 3. This indicates that all reads, with the exception of 1062r, can be used to study 16S rRNA gene based phylogeny. However, in most cases a scattering is observed for large NFL patristic distances, indicating a rather poor association between distant sequences in the SR and NFL trees. Table S1 in File S1 shows that correlations between SR and NFL trees fluctuate with different tree searches. These fluctuations are the combined effect of differences occurring in branch lengths between trees generated from different tree searches on NFL and SR libraries, which, as mentioned in the previous paragraph, can be related to the phylogenetic content of the reads.

**Table 3:** Overview of the research parameters that were applied in comparisons of short read and nearly full length sequence libraries.

Libraries	Variable region	PC patristic^a^	PC pairwise^a^	wRMSD^b^	Slope patristic^c^	Slope pairwise^c^	RF d	WRF1^e^	WRF2^f^	RF-WRF1	RF-WRF2
338f vs NFL	V3	0.687	0.68	0.019	1.46	1.01	1916	636.74	756.32	1279.26	1159.68
338r vs NFL	V2 partially	0.754	0.81	0.017	0.7	1.07	1916	613.3	743.78	1302.3	1171.82
518f vs NFL	V4	0.804	0.83	0.014	0.67	1.27	1797	601.73	742.95	1195.47	1054.25
518r vs NFL	V3	0.702	0.69	0.018	1.07	0.98	1914	637.43	754.86	1276.17	1158.74
799f vs NFL	V5	0.745	0.84	0.017	1.08	0.75	1938	641.39	755.13	1297.01	1183.27
799r vs NFL	V4 (almost complete)	0.787	0.83	0.015	0.58	1.32	1833	615.35	735.74	1217.45	1097.06
926f vs NFL	V6	0.692	0.72	0.019	1.17	1.05	2032	702.96	801.56	1329.44	1230.84
926r vs NFL	V5	0.729	0.84	0.017	0.82	1.04	1838	606.2	743.73	1232.2	1094.67
1062f vs NFL	V7 & V8 partially	0.82	0.78	0.014	0.59	0.64	1948	695.49	798.8	1252.51	1149.2
1062r vs NFL	V6	0.664	0.72	0.019	1.12	1.05	2015	643.48	758.94	1371.32	1255.86

a , PC =  Pearson Correlations, values presented are the means that were obtained from the different tree comparisons.

b , wRMSD =  Weighted Root Mean Square Deviations, values presented are the means that were obtained from the different tree comparisons.

c , slope was calculated for SR(1) versus NFL(1).

d , RF =  averaged Robinson Foulds distance between NFL and SR trees.

e , WRF1  =  averaged Weighted Robinson Foulds distance between NFL and SR trees based on the sum of the supports of the unique bipartitions.

f , WRF2 =  averaged Weighted Robinson Foulds distance between NFL and SR trees based on the sum of the supports of the unique bipartitions plus the difference of support values amongst the shared bipartitions.

A strong statistically significant negative correlation (R = −0.93, p<0.0005) indicated that the vCEED approach confirmed the results obtained with the Pearson Correlation method for comparisons between SR and NFL trees. The highest degree of fit was obtained for the 518f and 1062f libraries, closely followed by the 799r library.

#### With respect to tree topology

To find out whether branch length correlations were conform with consistency of the tree's branching pattern, RF and WRF distances were calculated between NFL and SR trees. The SR libraries that best conserved NFL tree topology were the 518f, 799r and 926r libraries (Table 3). The SR libraries that least conserved NFL tree topology were those targeting the V6 region, i.e. 1062r and 926f (Table 3). Despite the relatively large RF distances between NFL and 1062r SR trees, the WRF1 and WRF2 distances were relatively small, in the same range of 338f/NFL and 518f/NFL distances. This indicates that a large part of the bipartitions that are unique in the 1062r or NFL tree have a low support value. The 1062f trees, which had the lowest WRF values between trees generated from different tree searches amongst the SR libraries investigated (WRF1, Table 2), showed a relatively low conservation of NFL tree topology (RF, Table 3). Similarly, the WRF1 and WRF2 distances between 1062f SR trees and NFL trees were high (Table 3). These observations show that trees generated from the 1062f library did not conserve NFL topology.

**Figure 3:**
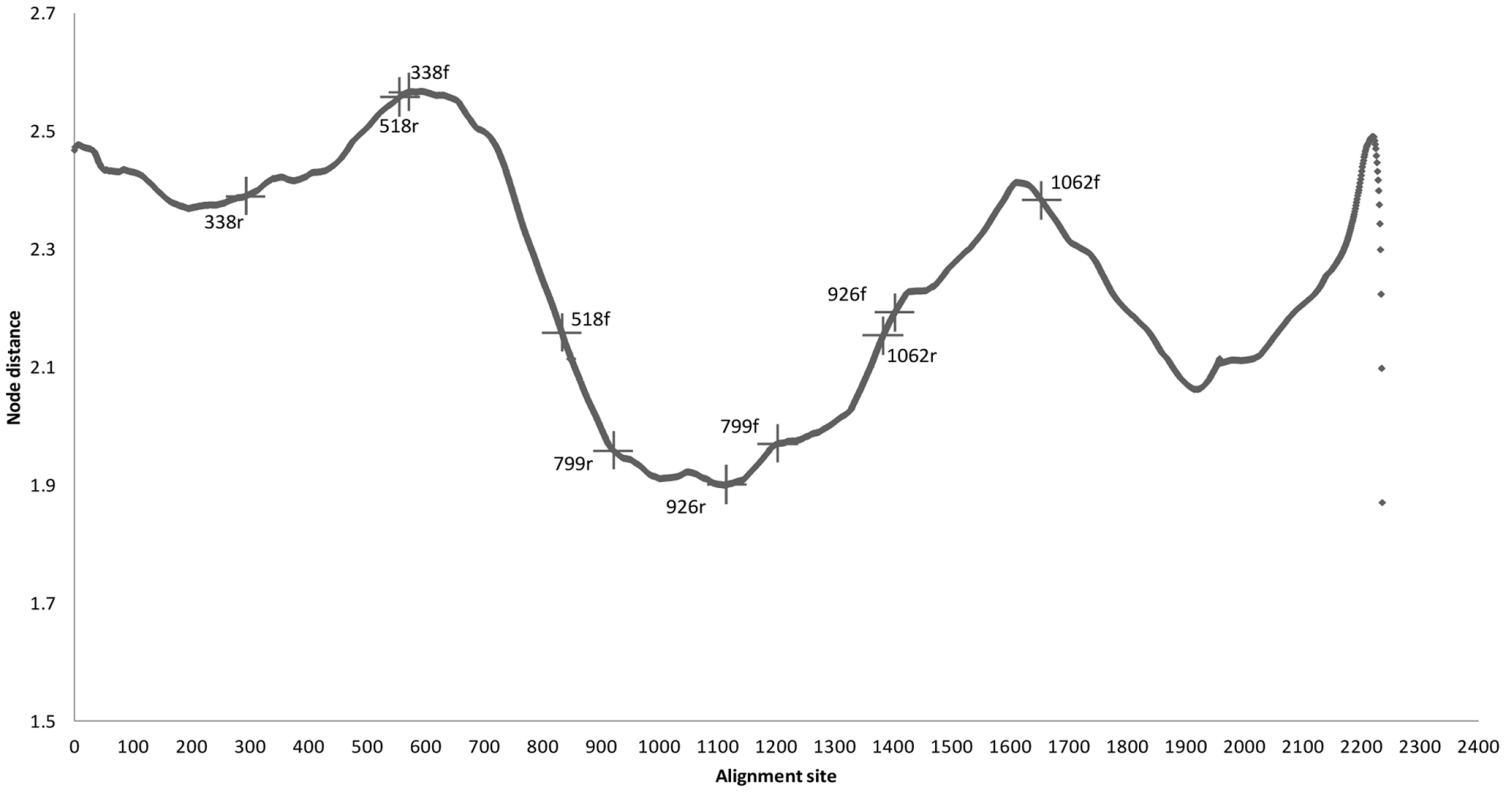
The result of the sliding window analysis on the nearly full length alignment. The size of the window was 280 bp, conform the length of the short read sequences. Plus signs indicate the points that cover the regions sequenced by the primers studied.

The sliding window analysis allowed quantifying the congruence of each alignment site with the overall NFL tree topology. The result of the analysis is given in Fig. 3. The alignment position (x-axis) marks the position of the first base within the sliding window; the node distance (y-axis) expresses the distance between the best tree generated from the NFL sequences and a tree modified starting from the NFL tree based on information as available from short read sequence data. The better the 280 bp window based modified tree correlates with the original NFL tree, the lower the distance in terms of nodes will be. The lower the node distance the more congruent the respective alignment site is to the overall tree topology. Fig. 3 shows that the best congruence with NFL tree topology was obtained for reads covering the V4, V5 and V6 regions of the 16S rRNA gene. The analysis also shows that amongst the V4 targeting reads, best congruence with NFL tree topology was obtained with 799r generated reads. Reads generated from the V2 and V3 targeting primers, as well as reads generated from the 1062f primer seemed to be less representative for NFL sequences with respect to tree topology.

### Relation between patristic distances in SR and NFL trees

The Pearson Correlation does not provide information about the extent to which patristic distances in the SR tree approximate corresponding distances in the NFL tree. To address this question we calculated the slope of the best-fitting line forced through the origin of the chart (Table 3). Reads generated from primers 338f, 926f, 1062r, 799f and 518r were found to generally overestimate branch-length distances, while reads generated from primers 926r, 338r, 518f, 1062f and 799r were found to generally underestimate branch-length distances. The 799f and 518r libraries approximated NFL patristic distances best.

### Resolving power of SR fragments

#### In relation to patristic NFL distances

The sizes of the error bars on the averaged SR distances (Fig. 2) are an indication for the resolving power of a SR fragment for a given normalized distance in the NFL tree. As mentioned in the methods section, branch lengths in the SR tree were averaged for each 0.01 distance unit interval in the NFL tree and the corresponding standard deviation on branch lengths in the SR tree was calculated. For a particular averaged NFL branch length, a high standard deviation indicates that the phylogenetic information within the reads did not allow to resolve the true branch lengths between all concerning pairs of sequences in the SR tree. In contrast to the Pearson Correlation, the standard deviation provides insight in the variation of patristic distances in the SR tree relative to a given normalized distance in the NFL tree. As such, it provides insight in the resolving power of the read for any normalized patristic distance in the NFL tree. The path of this standard deviation, plotted in function of the patristic distances in the NFL tree, is given for each read library in Figure S1 in File S1. In general, a scattering is observed at NFL patristic distances larger than 0.8, which is explained by the decreasing amount of patristic distances contributing to each averaged distance interval for larger distances. We should note that for interpretation of the standard deviation curve standard deviations corresponding to distances larger than 0.8 were not taken into account. The y-axis was set at a maximum value of 0.2 in order to gain more detail in the path of the standard deviation curve. Limiting this maximum value caused the loss of some non-informative outlier points at patristic distances larger than 0.8. A general trend is that the standard deviation increases with increasing NFL patristic distance. In some cases (i.e. 518f, 799f, 518r and 799r) the standard deviation reaches a maximum value at a certain NFL branch length, and then fluctuates around this maximum value for increasing patristic distances. This implies that the resolving power generally decreases for distant sequences, and in a number of cases varies around a constant minimum value from a specific NFL patristic distance forward. Libraries generated from the 338f, 518f, 518r, 799r and 1062f primers were found to generally have the lowest standard deviation over the complete range of NFL patristic distances, which means that these libraries have the highest resolving power over all NFL patristic distances. The 926f library peaked to the highest standard deviation amongst all libraries. In the special case of the 1062r library, the resolving power decreased with increasing NFL patristic distance to reach a minimum, but from that value forward increased for even more distant sequences.

#### In relation to pairwise NFL distances

Figure S2 in File S1 shows the standard deviation on the averaged pairwise SR distances in function of the pairwise distances in the NFL tree. Similar to the plots for patristic distances, a scattering is observed for normalized pairwise distances larger than 0.6. These points were not taken into account for interpretation. The y-axis was set at a maximum value of 0.2, which caused the loss of some non-informative outlier points. A general trend is that the standard deviation increases with increasing NFL distance. In the case of read 1062r, the standard deviation reaches a maximum value for an NFL distance of approximately 0.4, and then fluctuates around this maximum value for increasing patristic distances. These observations imply that, in general, the resolving power decreases for distant sequences, and in the special case of 1062r varies around a constant minimum value from a specific distance forward. Libraries generated from the 338f, 518f, 518r, 799r and 926r primers were found to generally have the lowest standard deviation over the range of NFL distances up to 0.6, meaning that these libraries have the highest resolving power over all NFL distances in question.

### OTU richness assessment in SR libraries based on pairwise distances

**Figure 4:**
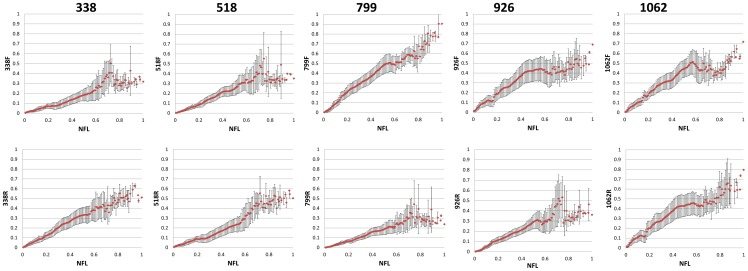
The Pearson Correlation between corresponding pairwise distances in trees generated from nearly full length (x-axis) and short read libraries (y-axis) for the different primers investigated. Pairwise distances were normalized to a maximum value of one prior to plotting.

The Pearson Correlation between pairwise distances in SR libraries and corresponding pairwise distances in their parent NFL library was never close to 1.00. The highest correlations were found for the 338r, 518f, 799f, 799r, 926r and 1062f reads (Fig. 4 **,** Table 3), confirming what was observed for patristic distance correlations between SR and NFL sequences. In each correlation plot (Fig. 4) we observe a strong correlation up to normalized pairwise distances of 0.5 to 0.6 on the x-axis. For larger distances there was some degree of scattering, depending on the library. This implies that for sequences with a high degree of similarity within a NFL library, the daughter SR sequences are proportionally similar within the SR library. However, this association is lost for sequences with a low degree of similarity. Since correlations do not provide any information about the extent to which pairwise distances between SR sequences approximate pairwise distances between their parent NFL sequences, we calculated the slope of the line of best fit forced through the origin in the NFL versus SR pairwise distance plots. Youssef et al. [7] found that the slope depends on the proportion of hypervariable, variable and conserved bases in the region of the 16 rRNA gene sequenced. Distances within the 338f and 518r libraries were found to be the best estimators of distances between NFL sequences, with slopes of 1.01 and 0.98 respectively (Table 3). Similarly, OTU richness calculated from the 518r and 338f libraries best approximated OTU richness calculated from NFL sequences (Table 2). However, no significant relationship was found between OTU richness calculated from the SR libraries, and the slope of the best fitting line forced through the origin (R = 0.64, 0.59 and 0.65 for OTU cut-offs of 0.01, 0.02 and 0.03 respectively). This was somehow unexpected, but could have been due to the fact that pairwise distances for OTU assignment were calculated using the Mothur software, while distance correlation plots were based on pairwise distances calculated in RAxML. It was shown previously that distance calculation method and parameters used have a significant effect on OTU richness [4]. Still, regardless of this discrepancy, the data shows a clear effect of the region sequenced on α-diversity in terms of OTU richness (Table 2). In each case there was an underestimation of OTUs compared to the NFL sequences. It is clear that these findings argue with the assumption frequently made that distances between short reads are representative for distances between full-length 16S rRNA gene sequences.

### Taxonomic assignment of SR sequences

Table 4 summarizes results on the taxonomic assignment performance of each SR library. Assignment performance was assessed by comparing identifications for each read within a SR library with identifications obtained for the parent NFL sequences in the NFL library. Taxonomic assignment was performed both at the phylum and genus level. The 518f library was found to generate the highest percentage of correct assignments at the genus level (80.15%), followed by the 338f, 799r and 518r libraries with 76.43%, 76.17% and 76% correct assignments respectively (Table 4). These observations confirm results obtained by Liu et al. [5] and Soergel et al. [6]. At the phylum level results were slightly different. The best assignments were obtained with the 518f, 799f, 799r, 926r, 338f and 518r libraries, all of which gave a comparably high number of correct assignments. Although the number of correct assignments obtained with the other SR libraries was lower, the difference was almost negligible. Short read sequences that were identified while the NFL sequence could not, were labeled false positives. The 799f library returned the smallest amount of false positive genus identifications, while the 926f and 1062r libraries returned the highest amount. At the phylum level, the number of false positive assignments was comparable for all libraries. Conversely, a number of SR sequences could not be assigned, while the NFL sequence was in fact assigned. Such SR sequences were labeled false negatives. Both at the genus and phylum level, the 518f library returned the lowest amount of false negatives while the 1062f library returned the highest amount. Based on these results it can be concluded that the 518f library is the best target for assignment of short reads. With the exception of false positives (for which it scored last but one), the 518f library scored best for the different criteria for both genus and phylum level identifications.

**Table 4:** Taxonomic assignment performance of short read sequences.

Taxonomic assignment	PHYLUM (%)										GENUS (%)									
	338f	338r	518f	518r	799f	799r	926f	926r	1062f	1062r	338f	338r	518f	518r	799f	799r	926f	926r	1062f	1062r
**Correct assignments by read (including unclassified NFL reads)** a	98.55	97.79	98.98	98.47	98.89	98.81	97.45	98.72	97.11	97.45	76.43	72.43	80.17	76	64.09	76.17	71.57	70.38	63.66	72.43
**Correct assignments by read (excluding unclassified NFL reads)** a	98.72	98.03	99.14	98.72	99.06	98.97	97.6	98.89	97.26	97.6	75.37	71.02	79.54	74.81	61.57	75.09	70.37	68.61	61.39	71.3
**False positive reads** b	0.17	0.26	0.17	0.26	0.17	0.17	0.17	0.17	0.17	0.17	0.94	0.94	1.02	0.85	0.6	0.94	1.19	0.77	0.85	1.19
**Unclassified reads (total)**	1.36	1.79	1.02	1.28	1.19	1.19	2.38	1.36	2.89	2.47	27.91	32.94	24.51	28.17	37.28	28.68	30.3	32.09	40	29.87
**False negative reads** c	1.02	1.53	0.68	1.02	0.85	0.85	2.04	1.02	2.55	2.13	20.77	25.79	17.45	20.94	29.79	21.53	23.4	24.77	32.77	22.98
**Unclassified NFL reads**	0.34	0.26	0.34	0.26	0.34	0.34	0.34	0.34	0.34	0.34	7.15	7.15	7.06	7.23	7.49	7.15	6.89	7.32	7.23	6.89

a , percentage of reads that were assigned to the same genus/phylum as in the NFL library.

b , reads that were classified in the SR library but unclassified in the NFL library.

c , percentage of reads that were classified in the NFL library but unclassified in the SR library.

## Discussion

The aim of this research was to analyze the suitability of commonly used, published primers targeting dispersed regions of the bacterial 16S rRNA gene for short read sequencing. The study targets different aspects that each are involved in data interpretation. We started by calculating primer coverage rates for each of the primers analyzed, and continued with the phylogenetic information that is contained within short reads. Subsequently, the relation between pairwise distances in NFL and SR sequence libraries was studied to assess the effect on OTU richness. We ended by investigating the taxonomic assignments obtained with each of the SR libraries. In order to do so, we constructed a sequence library composed of 1175 sequences, which served as a representative substitute of the SILVA SSU database. The choice to work with this representative library was motivated by the fact that we did not want to focus on a specific environment, which is inherently biased towards specific taxonomic groups, but instead we aimed at making our results applicable for divergent taxa, and consequently for a variety of environments.

The methodology used allows for a thorough evaluation of the scientific outcome that is obtained with sequencing short read fragments generated from primers targeting dispersed regions of the 16S rRNA gene. For the outline of this study, we started by following the reasoning of Jeraldo and colleagues [10] who focused on *de novo* synthesis of phylogenetic trees from short reads to study the implications of information loss which is inherent to sequencing short fragments of the 16S rRNA gene. We extended their well designed approach by checking whether short reads can be used to infer 16S rRNA gene based phylogeny and by assessing whether short reads are reliable estimators of relationships between their parent NFL sequences in terms of patristic distances. Insight in the resolving power of short read fragments for any patristic or pairwise distance between NFL sequences was obtained from standard deviations on averaged short read distances. Next, the relation between pairwise distances between short read fragments and pairwise distances between NFL sequences was studied. This information was used to perceive the effect of sequencing different regions of the 16S rRNA gene on OTU richness and taxonomic assignment accuracy. Additionally the coverage rates of the primers were calculated based on sequences in public 16S rRNA gene databases. We acknowledge the fact that these databases are composed of sequences that were obtained from amplicon sequencing, which makes the results obtained prone to PCR amplification bias. Inclusion of metagenomic data, as performed by Mao and colleagues [11], would have given a superior picture. However, as the emphasis of this study was on phylogenetic and taxonomic information, we considered this extension of primer coverage rate beyond the scope of this study.

Our results show that the 518f reads that target the V4 region of the bacterial 16S rRNA gene were generally most informative. The correlation value of 0.97 (and the high degree of fit) that was obtained after comparing 518f trees from different tree searches is a very optimistic approximation to the upper limit of 0.98, and indicates the high phylogenetic content of these reads. High correlation values were maintained with an increasing number of tree searches, indicating that the trees generated were very reproducible with respect to patristic distances. Although 518f reads tended to underestimate patristic distances in ML trees, they were found to best reflect 16S rRNA gene based phylogenetic relationships with good resolving power. The 518f reads were found to score best for most of the criteria investigated to assess taxonomic assignment performance. However, nonetheless a high correlation (and degree of fit) was observed between pairwise distances in SR libraries and corresponding pairwise distances in the parent NFL library, reads were not the best estimators of pairwise distances between NFL sequences (cf. slope). This had its effect on OTU richness, for which the 518r and 338f libraries were found to perform better. Furthermore, primer coverage rates showed that the 518f/r primer is not specific for bacterial 16S rRNA, which implies that contamination with eukaryotic and archaeal 16S rRNA genes may occur.

Since 799r reads also target the V4-region of the 16S rRNA gene, it was not surprising that the primer in question was also found to be a promising instrument for short read sequencing studies. The Pearson Correlation and the degree of fit between patristic distances that were extracted from SR and NFL trees were higher for reads generated with the 799r primer than with the 799f primer. The same was observed for multiple tree searches on the same library. The Pearson Correlation between pairwise distances in the 799f library and the NFL library was similar to the Pearson Correlation between pairwise distances in the 799r library and the NFL library. The high correlation values that were obtained in both cases indicated that both libraries reflect similarities between NFL sequences. Sizes of the error bars in both the patristic and pairwise correlation plots were generally larger in 799f generated reads than in 799r generated reads, indicating a higher resolving power of the 799r reads. The slope of the best fitting line through the origin was 1.08 for the 799f primer, which is a good approximation of NFL patristic distances. The slope calculated for the 799r library, however, was only 0.58, indicating that in general branch lengths were 42% shorter. The 799r reads tended to overestimate differences between sequences, while the 799f reads tended to underestimate differences, with a clear effect on α-diversity. Of both libraries, OTU richness in the 799r library was a better estimator of OTU richness in the NFL library. In terms of taxonomic assignment of SR sequences at the phylum level, performance was comparable for the 799f and 799r libraries for the different criteria investigated. However, at the genus level the 799r library generally performed better than the 799f library.

Our results illustrate that the 1062f/r primer had the highest coverage rate over the 29 phyla studied. Therefore, this primer is most likely to target the broadest bacterial diversity amongst the primers investigated. However, the 518f library scored best for most of the criteria that allow measuring to which extent the information obtained from short reads is representative for their parent full length sequences. In some cases the use of the 799f/r primer is recommended in order to avoid the interference caused by co-extracted host organelle DNA. For such cases, the results obtained show that the 799f/r primer is best used in the reverse direction in order to optimally exploit the information contained within short sequencing reads. However, it was mentioned earlier that in order to exclude the interference of host derived mitochondrial sequences the primer should be used in the forward direction. The consideration between information loss due to the presence of mitochondrial sequences when using the primer in the reverse direction, and information loss due to the less informative region sequenced in the forward direction is a decision that should be driven by the aims of the research.

## Supporting Information

Files S1 **Contains Figure S1 and S2 and Table** **S1.** Figure S1 in File S1: The resolving power (y-axis) of short reads for any normalized patristic distance in the NFL tree (x-axis). Figure S2 in File S1: The resolving power (y-axis) of short reads for any normalized pairwise distance in the NFL tree (x-axis). Table S1 in File S1: Overview of the research parameters that were applied in comparisons of short read and nearly full length sequence libraries – individual values for each of the tree comparisons.
(XLSX)
